# Screening for Barrett’s Oesophagus: Are We Ready for it?

**DOI:** 10.1007/s11938-021-00342-1

**Published:** 2021-03-16

**Authors:** Aisha Yusuf, Rebecca C. Fitzgerald

**Affiliations:** Medical Research Council Cancer Unit, Hutchison/Medical Research Council Research Centre, University of Cambridge, Cambridge, CB2 0XZ United Kingdom

**Keywords:** Barrett’s oeosphagus, Oesophageal adenocarcinoma, Screening, Endoscopy, Cytopsonge, Early detection of cancer

## Abstract

**Purpose of review:**

The targeted approach adopted for Barrett’s oesophagus (BO) screening is sub-optimal considering the large proportion of BO cases that are currently missed. We reviewed the literature highlighting recent technological advancements in efforts to counteract this challenge. We also provided insights into strategies that can improve the outcomes from current BO screening practises.

**Recent findings:**

The standard method for BO detection, endoscopy, is invasive and expensive and therefore inappropriate for mass screening. On the other hand, endoscopy is more cost-effective for screening a high-risk population. A consensus has however not been reached on who should be screened. Risk prediction algorithms have been tested as an enrichment pre-screening tool reporting modest AUC’s but require more prospective evaluation studies. Less invasive endoscopy methods like trans-nasal endoscopy, oesophageal capsule endsocopy and non-endoscopic cell collection devices like the Cytosponge coupled with biomarker analysis have shown promise in BO detection with randomised clinical trial evidence.

**Summary:**

A three-tier precision cancer programme whereby risk prediction algorithms and non-endoscopic minimally invasive cell collection devices are used to triage test a wider pool of individuals may improve the detection rate of current screening practises with minimal cost implications.

## Introduction

Oesophageal adenocarcinoma (OAC) is a poor prognosis cancer [1] that is often preceded by histologically defined premalignant lesions called Barrett’s oesophagus (BO). This provides excellent opportunities for screening (identification of the disease) and surveillance (disease monitoring). Pre-emptive screening and surveillance programmes could have a big impact on outcomes, survival and comorbidity associated with treatment, since outpatient-based endoscopic therapies for premalignant lesions can prevent invasive disease. To date the mainstay for diagnosis and monitoring has been endoscopic-based technology [2]; however, the relative expensive and invasive natures of oesophago-gastro-duodenoscopy (OGD) impede the effective implementation of using this diagnostic modality in a population-wide screening programme. This has become even more apparent with the pressures on diagnostic services during the coronavirus pandemic. These limitations have thus warranted development of other less invasive, more affordable screening tools for mass screening as well as for surveillance and diagnostic triage.

## Challenges associated with current screening guidelines

The precursor lesion for OAC is non-dysplastic BO (NDBO) which can subsequently progress to low-grade dysplasia (LGD), high-grade dysplasia (HGD) and eventually to OAC [3]. The annual risk of progression to OAC steadily increases from 0.1 to 0.5% in NDBO to 6% or more in HGD [4–7]. In some cases, progression can be rapid without opportunity to diagnose intermediate dysplastic steps. However, since population-based studies show that BO diagnosis prior to OAC detection decreases disease mortality [8], and since there has been such an improvement in outpatient, endoscopic therapies for early disease, there is a strong rationale for programmes to improve detection of BO.

A recent systematic review and meta-analysis of papers from the past 10 years reported that 11.8% of OAC cases had a known diagnosis of BO [9••], even taking into account increasing endoscopy volumes [6, 10–12]. There are several likely explanations behind the substantial numbers of missed screening opportunities for patients with undiagnosed BO. The societal screening guidelines are recommendations, but national public health agencies have not enforced OGD-based mass screening owing to the poor cost benefits and potential morbidity associated with using endoscopy as a screening tool for a relatively uncommon disease. This is especially the case when compared to the higher prevalence of other cancer types like breast and colon. Although there is consensus on the role of BO as a risk factor for OAC and the need for some form of screening, there are different views on “who”, “when” and “how” to screen. Furthermore, these guidelines are not always supported by high-quality evidence and are rather based on expert opinions [13]. Moreover, the cost efficacy and improved mortality rate of this targeted screening approach has never been evaluated.

In terms of who to screen, guidelines from all societies thus restrict screening to more targeted high-risk populations [2, 14–18]. Both genetic and non-genetic risk factors have been identified for OAC. However, polygenic risk scores are not yet being applied clinically and family history is used as a proxy measure of inherited genetic risk [19]. Regarding non-genetic factors, chronic and frequent gastroesophageal reflux disease (GORD) is the most significant and well-studied. A landmark population-based case-control study demonstrated that patients with GORD had a significantly greater risk of developing OAC as compared with asymptomatic individuals with an odds ratio of 7.7 (95% CI 5.3–11.4) in patients with recurrent reflux symptoms and 43.5 (95% CI 18.3–103.5) amongst patients with chronic and long-standing symptoms (>20years) [20]. Historically, chronic GORD has thus been the sole symptom used for initiating entry into BO/OAC endoscopic screening programmes. However, approximately 40% of patients with OAC do not present with chronic reflux symptoms despite having severe GORD [21]. This so-called “silent reflux” is thought to be explained by a reduction in oesophageal sensitivity to acid refluxate following metaplastic conversion to BO [22]. Nevertheless, using GORD as a screening criterion has its indisputable merits as modelling on the adult US population indicates that screening test would be most impactful in symptomatic GORD patients since they are responsible for 52% of all cancer cases and necessitate screening only 20% of the population [23]. In this scenario, the term “diagnostic test” may be a more fitting word to use than “screening” since only symptomatic persons are being tested unless individuals are invited pro-actively dependent on a search of prescribing databases for example.

The cost benefits of targeted screening have led to the modification of recommendations in current society guidelines to consider additional risk factors like family history, central adiposity, Caucasian ethnicity and male sex [19, 24–26]. There is also concern about the risks of over-diagnosis and over-treatment since the use of symptoms and risk factors is a blunt tool to predict BO and is difficult to implement, and it remains the case that the majority of patients diagnosed will not progress to OAC. Hence, combinatorial risk models are required taking into account the feasibility of applying an algorithm in clinical practise.

## Risk prediction models

An alternative two-stage screening approach has been sought whereby a subset of patients identified as having an increased probability of having BO are screened in a primary care setting with a less expensive and less invasive test and then referred for endoscopy confirmation. Statistical risk prediction models using widely available and easy to obtain symptoms and risk factors have been developed to estimate the absolute risk that an individual has BO or would develop OAC [27]. For example, a BO prediction model based on electronic health records (EHR) data including gastroesophageal reflux disease, sex, body mass index and ever-smoker status was shown to identify BO patients with a modest accuracy reporting an AUC of 0.71 (95% CI 0.64–0.77) [28]. With risk prediction tools, there is an added potential opportunity for self-assessment whereby a patient can produce a personalised risk profile for BO using a web-based application. Despite the accessibility-related benefits and cost-effectiveness, none of these models is ideal having reported AUCs ranging from 0.61 to 0.75 [27, 29]. Rosenfeld et al. recently developed and validated a machine learning-based risk prediction model for BO (called MARK-BE) using a comprehensive panel of 8 features observed to be significantly associated with increased risk of BO (age, sex, cigarette smoking, waist circumference, frequency of stomach pain, duration of heartburn and acidic taste and taking acid suppressants) [30•]. The MARK-BE model appears to be more robust than other BO risk prediction algorithms published previously due to the large datasets used in the study and the cross validation performed. The model was trained internally with a dataset (collected from BEST 2 case-control screening study (ISRCTN 12730505)—training dataset (*n* = 776), testing dataset (*n* = 523)) and subsequently validated externally in an independent dataset (collected from the BOOST case-control study (ISRCTN 58235785) *n* = 398) reporting an area under the receiver-operator curve (AUC) of 0.81 (95% CI 0.74–0.84, sensitivity set at 90% and specificity of 58%). Although these results look promising, the findings have to be validated in a primary care setting and the optimal thresholds of risk at which screening is warranted using this model have to be determined.

**Fig. 1.**
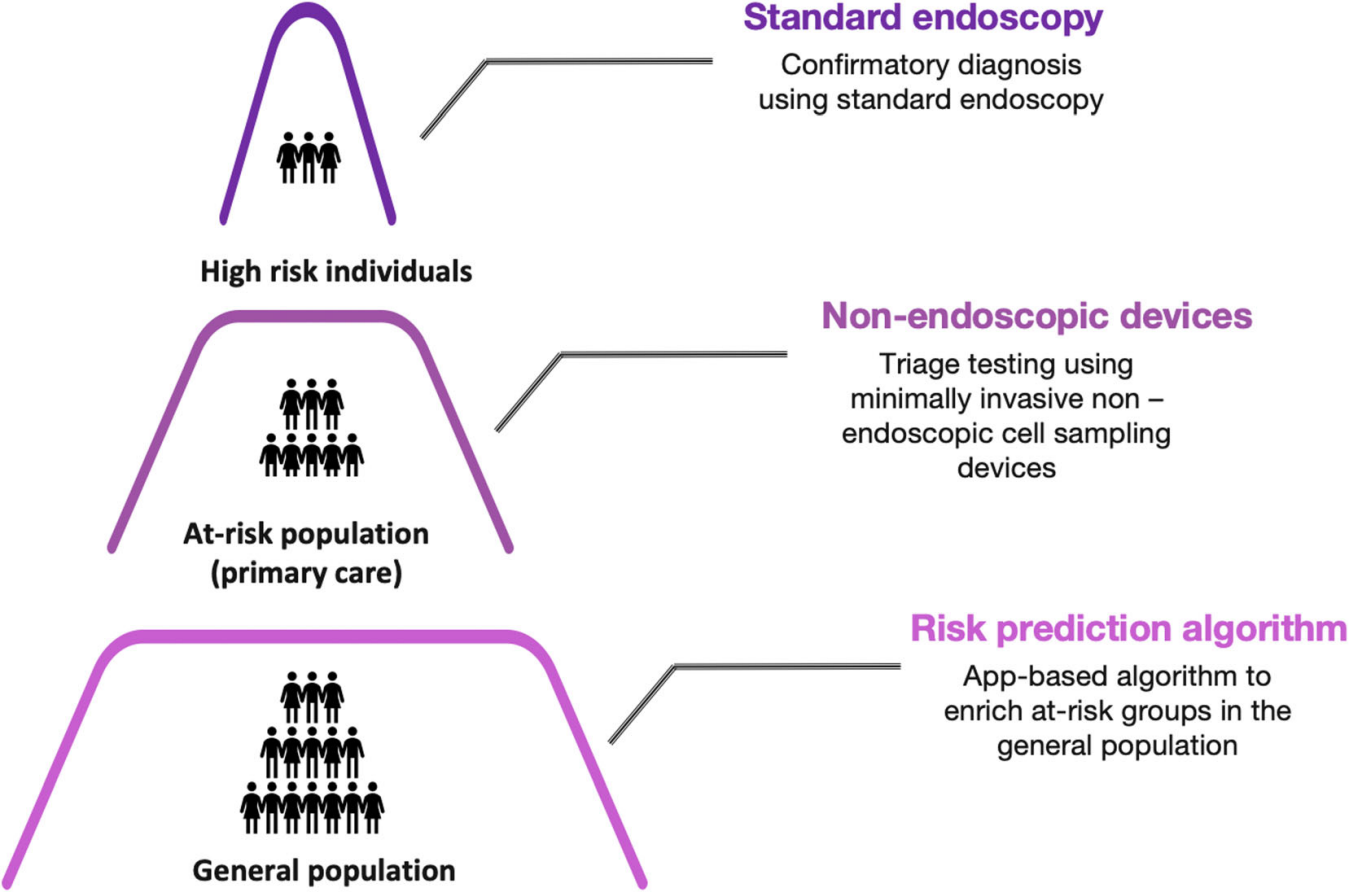
Three-tier precision cancer prevention strategy.

For the current screening paradigm to be successful in OAC prevention, we need to identify the “at-risk” population with greater precision and/or develop other safe, economically viable, minimally invasive screening techniques that can allow effective, systematic screening for BO. For instance, an attractive strategy of “precision cancer prevention” has been proposed whereby a 5-tier system is used to stratify individuals into more precise risk groups with each group being allocated risk-appropriate screening and management options [23]. For example, individuals belonging to the lowest risk strata would require minimal intervention and simple cost-effective screening tools whereas high-risk groups would be eligible for more precise but also more invasive and expensive techniques (Fig. 1). Such a comprehensive and tailored screening protocol would not only incorporate a wider population base, including individuals who are dismissed by current guidelines, but also leverage the use of confirmatory endoscopy only in situations where it is warranted thus saving resources.

## Screening with the use of imaging techniques

As noted, although white light endoscopy remains the cornerstone of the current BO screening practise, it is unlikely to be suitable for mass screening and even routine screening of at-risk individuals because of the direct and indirect cost burden associated with sedation [31] and side effects [32], albeit a highly safe and generally well-tolerated procedure. Trans-nasal endoscopy (TNE) is an alternative endoscopic technique that uses ultra-thin endoscopes (outer diameter <6mm) performed in the sitting position with the instrument inserted through the nose for endoscopic imaging of the distal oesophagus. TNE reduces gagging and vomiting risk thus improving patient tolerability making it more suited for implementation in primary care [33–35]. It has also been used in mobile research vans for oesophageal assessment and screening for BO in the community [36]. In expert hands, TNE is as sensitive and specific as standard OGD in detecting BO in at-risk population [37]. Result from a meta-analysis of 34 studies with 6659 patients showed a comparable technical success rate (94.0% and 97.8%, respectively) from both TNE and OGD with a significantly higher proportion of patients preferring the former method [38]. TNE is thus recommended in recent guidelines for BO screening due to its comparable diagnostic accuracy, improved patient tolerability and lower direct and indirect cost. This technique has however not been widely embraced, and this may reflect its limited availability, since it requires investment in specialised equipment, decontamination facilities and trained operators, as well as attitudinal barriers amongst clinicians and patients. Further modifications have been carried out on the original TNE to overcome these limitations. The EG Scan TM II (Intromedic Ltd., Seoul, South Korea) is a second-generation TNE device comprising a disposable trans-nasal video esophagoscope with a compact image processing unit. In a tertiary population, with an increased BO prevalence, the EG Scan has been shown to be feasible, safe and capable of detecting BO of any length with a sensitivity value of 0.90 (95% CI, 0.83–0.96) and a specificity value of 0.91 (95% CI, 0.82–0.96), in comparison to conventional esophagogastroduodenoscopy [39]. It also had a high acceptability rate amongst the 89% of participants in the study. Of note, TNE devices have little to no biopsy sampling capabilities and cannot be used for endoscopic therapy. Since biopsy-based diagnosis currently holds the most accurate clinical diagnostic value for BO and OAC, such tools may thus only be useful in two-step screening programme where they could be used for preliminary triage testing in order to enrich the population and then subject individuals with suspected BO to conventional endoscopy for acquisition of multiple, larger, surveillance biopsies to detect dysplasia and enable risk stratification and treatment.

Oesophageal capsule endoscopy, an alternative to the TNE technology, comprises a wireless pill-sized capsule enclosed with a camera, battery and radio transmitter. Images are transmitted by the radio to a digital receiver worn on the waist of the patient and subsequently downloaded into a computer (approximately 8 hrs later) for analysis. Like TNE, OCE does not require sedation and can be carried out in an office setting. The advantage of OCE over conventional TNE is that it is a single-use device obviating the need for equipment reprocessing. The traditional OCE technology has however been reported to have suboptimal diagnostic accuracy with rapid oesophageal transit times which could hinder thorough inspection of the oesophagus [40]. Therefore, newer versions of OCE have been developed so called wireless capsule endoscopy in efforts to overcome problems of rapid oesophageal transit and to allow prolonged imaging and inspection of suspected areas. An example of such innovative modification is the detachable magnetically controlled capsule endoscopy [41]. Tethered capsule endomicroscopy is another variant of OCE that uses optical frequency domain imaging technology [42] to capture 3-dimensional, cross-sectional microscopic images of the entire oesophagus. Subtle tissue microscopic subsurface information that can otherwise be missed by endoscopy is thus provided without requiring microscopic assessment of sampled biopsies. Studies on TCE are promising as the method has been shown to be safe, capable of distinguishing patients with and without BO and feasible in primary care setting for screening BO [43, 44].

Despite great strides made with the OCE technology, larger prospective studies in the relevant primary care population are needed to assess cost-effectiveness and the diagnostic accuracy of these methods for the identification and surveillance of preneoplastic or neoplastic oesophageal lesions.

## Non-endoscopic cell collection devices coupled with biomarkers—Cytosponge, EsophaCap and EsoCheck

An alternative strategy to the use of imaging for BO screening would be to sample cells along the oesophagus using minimally invasive non-endoscopic devices and subsequent analysis of molecular aberrations and/or cytopathologic features associated with BO on the cells collected. Non-endoscopic cytological methods have historically been investigated as screening and early detection tools for oesophageal squamous cell cancer in high prevalence countries particularly China. Although these minimally invasive techniques such as brush cytology, mesh and balloon samplers were safe in the majority of study subjects with minimal adverse effects reported, none of these historic studies lead to implementation of the methods established in population-based screening programme owing to poor test sensitivity of traditional cytology. This led the Fitzgerald lab to develop an alternative minimally invasive cell collection device that would retrieve a higher number of cells in an operator independent manner and to augment cytological examination with biomarkers. Although this approach has not yet been recommended in current society guidelines, the recent technical advances and randomised trial data are moving towards clinical implementation.

The main non-endoscopic cell collection devices currently at different stages of development include the Cytosponge (Covidien products, Medtronic, Minneapolis), EsophaCap (Capnostics, Doylestown) and EsoCheck (Lucid Diagnostics, New York) with each technology having its specific strengths and limitations (Table 1). These devices were developed with the intent to be used for large-scale triage testing in the diagnostic or screening setting, and as such, the procedure is performed by a trained nurse or physician, and a positive result test would warrant a follow-up confirmatory diagnosis via the standard OGD method.

**Table 1:** Key differences in the three non-endoscopic cell collection devices studied for the detection of Barrett’s oesophagus

Test	Cytosponge-TFF3	EsophaCap with 5 gene methylation panel	EsoCheck with EsoGuard (2 gene methylation panel)
Device type	Pan-oesophageal sampling device	Pan oesophageal sampling device	Targeted distal oesophagus sampling device
Characteristics	Not operator dependent	Not operator dependent	Operator-dependent balloon inflation and deflation
Biomarkers	TFF3–IHC with potential for AI-assisted reporting [45, 46]	MDMs: VAV3, ZNF682, NDRG4, FER1L4, and ZNF568 [47]	MDMs: VIM and CCNA1 [48]
Safety record	•Detachment rate •1/2672 (<0.1%) [49] •1/1654 (<0.1%) [50••] •Minor bleeding: 1/2672 (<0.1%) [49] •Sore throat : 63/1654 (4%) [50••]	5/268 (2%) reported AE-1 tether detachment and 4 other events [47]	No AE recorded, and data on safety is limited [48]
Evidence trial type	•Multicentre cohort study in primary care (BEST1) [51] •A multicentre case control study—11 UK hospitals (BEST2) [52] •RCT (cluster and individual randomised) in >13,000 individuals in primary care (BEST3) [50••]	Multisite case-control study—three tertiary care centres and 1 community hospital in the US [47]	Non-randomised observational study—1 tertiary care tertiary care institution in the US [48]
Sample size (*n*), sensitivity, and specificity	BEST2 study: 1110 [52] (463 controls, 647 BO), sensitivity = 79.9%; 87.2% (≥ 3 cm BO), (per protocol including inadequate sampling), and specificity = 92.4%	295 (89 controls, 112 BO, 67 indeterminate) [47]**,** sensitivity = 92%, and specificity = 94%	156 (36 controls, 42 BO) [48], sensitivity = 90.3%, and specificity = 91.7%
Overall acceptability score (10-scale grading)	High score most acceptable 6 (IQR 5–8), *n*= 2418 9 (IQR 8–10), *n*=1654	Low score most acceptable 2 (IQR 0, 4), *n*=268	Low score most acceptable 1–2, *n*=92
RCT evidence	Yes Primary endpoint: detection Barrett’s increased with rate ratio 10.6 (95% CI 6.0–18.8) compared with usual care, *p*<0.0001 [50••]	No	No
Regulatory approval for device and assay	CE-marked—yes (device only) FDA 510(k)—yes	CE-marked—yes (device only) FDA 510(k)—not yet	CE-marked—not yet FDA 510(k)—yes
Other trials in progress	Observational, multicentre implementation research study (ISRCTN91655550)	Case-control study, *n*= 2500 (ClinicalTrials.gov Identifier: NCT04214119)	Multicentre single-arm study, *n*= 1000 (ClinicalTrials.gov Identifier: NCT04293458)

*IHC* immunohistochemistry, *MDMs* methylated DNA markers, *AE* adverse effect, *OGD* oesophagogastroduodenoscopy, *BO* Barrett’s oesophagus, *BEST* Barrett’s oesophagus screening trial

The Cytosponge comprises a 30-mm polyurethane sponge, compressed within a gelatine capsule about the size of a vitamin pill attached to a string. Once the patient swallows this capsule and it reaches the gastric cardia, the tightly packed capsule opens up to form a sponge. Cells lining the entire length of the oesophagus and oropharynx are collected when the Cytosponge is retrieved by pulling on the attached string. Similar to the Cytosponge, the EsophaCap is also a sponge on string device although slightly smaller and softer than the Cytosponge but likewise comprises a swallowable encapsulated sponge attached to a tether. The EsoCheck on the other hand is a swallowable balloon-based oesophageal sampling device comprising of a collapsible encapsulated balloon attached to a thin silicone catheter which is connected to a syringe. When the operator identifies the pressure change of the lower oesophageal sphincter the balloon is inflated by injecting air via the catheter, the balloon is gently withdrawn into the distal oesophagus sampling cells from 5–6cm above the GOJ. The device is designed to selectively target the distal oesophagus for sampling as subsequent deflation of the balloon through the catheter results in its inversion back into the capsule thus protecting the acquired bio-sample from further dilution or potential contamination by proximal oesophagus during the capsule retrieval process through the mouth. This more targeted sampling with EsoCheck increases the signal-to-background-noise ratio. However, this feature makes the sample acquisition process more operator dependent. The Cytosponge mesh has a more abrasive surface than the balloon thus allowing deeper samples to be collected. Oesophageal cytology cells captured by these devices are analysed using biomarkers ascertained for their sensitivity and specificity for BO and with the potential to add biomarkers for dysplasia detection.

Although samples collected via the Cytosponge can be assayed for several biomarkers including methylation [53], multigene next-generation sequencing panels [54], and microRNAs [55] useful for screening and surveillance, the current modus operandi for detecting BO from specimens obtained using this device is immunohistochemical staining of Trefoil Factor Protein 3 (TFF3) which is an indicator of intestinal metaplasia (the histopathological feature of BO). The slide-based technique permits the identification of gastric columnar cells as a quality control metric to ensure that the sampling device reached the stomach. In addition, other morphological features can be assessed including atypia indicative of dysplasia and inflammation, as well as application of additional biomarkers for p53 aberration and an immune-histochemical surrogate for copy number [52, 56]. The other two cell sampling technologies use BO-related methylated DNA biomarkers (MDMs), which unlike immunohistochemical staining, do not require subjective interpretation by a pathologist since MDMs can be scalable for automated high throughput testing. Moreover, a methylation-based circulating biomarker, (Epi proColon, Epigenomics) was approved by the U.S. Food and Drug Administration for colorectal cancer screening in 2016 [57].

### Evidence for Cytosponge-TFF3

Stepwise clinical trial evidence has been accrued for the Cytosponge-TFF3 procedure to determine safety, acceptability and efficacy. A multicentre case control study demonstrated a specificity for diagnosing BE of 92.4% (95% CI 89.5–94.7%) with a sensitivity of 79.9% (95% CI 76.4–83.0%), increasing to 87.2% (95% CI 83.0–90.6%) for patients with ≥3 cm of circumferential BO, known to confer a higher cancer risk. This was a per protocol analysis that included cases in whom the device did not reach the stomach. The sensitivity was >90% when columnar cells were present on the sample, and in the subsequent BEST3 trial, a repeat Cytsoponge test was recommended in this situation [58]. The BEST3 randomised controlled screening trial has shown that the systematic offer of this test to individuals with heartburn symptoms results in a 10-fold increase in the diagnosis of BO compared to usual care which entailed endoscopy as deemed necessary by the general practitioner [50••]. Furthermore, a comparative modelling study highlighted the cost-effectiveness of using Cytosponge to screen symptomatic GORD patients followed by confirmatory endoscopy-based diagnosis in Cytosponge positive cases having estimated a 27–29% reduction in screening cost using this approach in comparison to screening with endoscopy alone [59]. Iqbal et al. conducted an independent systematic review of 13 relevant studies to assess the efficacy and safety of the Cytosponge against the endoscopy/biopsy gold standard and reported promising results [60]. A recent systematic review of 5 large prospective trials evaluating Cytosponge performance in 2672 procedures showed the device to be safe and well-tolerated amongst patients with only 2 resolvable serious adverse events denoted to be directly attributed to the device and most patients ( 91.1% ) swallowed the Cytosponge, most on the first attempt (90.1%) [49]. The favourable safety profile and relative ease of the Cytosponge procedure in comparison to endoscopy makes it a promising screening tool in primary care setting. An implementation study, called DELTA, is currently underway in the UK to establish the practical steps for introduction into routine clinical care and to further evaluate cost-effectiveness and patient preferences to maximise uptake.

### EsophaCap biomarker

The EsophaCap was first tested in a pilot trial that reported the feasibility of using a 2-MDM model (vav guanine nucleotide exchange factor 3-VAV3 and zinc finger protein 682-ZNF682) with the EsophaCap to diagnose BO achieving 100% sensitivity and specificity in a highly enriched population comprising 20 BO patients 10 of whom had dysplasia and 20 controls [61]. No major complications were observed and only 32% of patients who swallowed the capsule had minimal abrasion without bleeding. The same group went on to conduct a multisite case-control study comprising 295 patients across 2 geographically distinct tertiary care centres and 1 community hospital in the United States (US) to assess the accuracy of the pre-established MDMs in a separate cohort [47]. They used a recently validated commercial grade assay (target enrichment long-probe quantitative amplified signal (TELQAS)) to achieve this [62] . In this study, 91% of consented participants swallowed the capsule of which 112 were categorised as BO cases, 89 as controls and 67 as indeterminate. The indeterminate group was studied separately following the primary analysis on cases and controls. VAV3 and ZNF682—the two MDMs discovered in the pilot trial—were included along with 3 additional markers NDRG4, FER1L4, and ZNF568 yielding a sensitivity and specificity of 92% (95% CI 85–96%) and 94% (95% CI 87–98%), respectively. Five patients (7%) encountered technical issues including device intolerance and tether detachment during the administration of the device and 1 inadequate DNA sample was reported. The procedure was nevertheless successfully undertaken by non-physicians in most study subjects, and the device was well-tolerated (median [interquartile range] tolerability 2 [0, 4] on 10-scale grading) with 248 study participants (94%) preferring the EsophaCap over OGD. Although the model accuracy was not influenced by age, sex or smoking history, as expected BO length altered the performance with four short segment, non-dysplastic BE cases missed by the model. Furthermore, no statistically significant difference was reported between the sensitivity of the 5-marker panel for non-dysplastic BO and dysplastic BP. A case-control study with 2500 participants in the US is ongoing to identify optimal DNA methylation biomarkers for the early detection of BO, oesophageal carcinoma and gastric cancer via EsophaCap-derived specimens (ClinicalTrials.gov Identifier: NCT04214119).

### EsoCheck biomarker

The EsoCheck device has been investigated with a two-marker MDM panel vimentin (VIM) and cyclin-A1 (CCNA1). A pilot trial conducted across patients (36 controls, 42 BO cases (31 non-dysplastic, 6 LGD, 4 HGD and 1 indefinite for dysplasia) and 8 with EAC or junctional cancers) reported a sensitivity and specificity of 90.3% and 91.7% for BO detection [48]. Although the device was generally well-tolerated, 18% of study participants could not swallow the device and the investigators reported poor DNA yield in an additional 9% who were excluded from the analysis. Modifications in the design of the original EsoCheck device was shown to improve the device’s failure to swallow rate and increased DNA yield for diagnosing BO/OAC [63]. In comparison with OGD, the efficacy of the newer generation EsoCheck device in combination with 2-marker MDM panel assay for the diagnosis of BO in at risk screening population is currently being tested in a multicentre single-arm study with a sample size of 1000 subjects in the US (ClinicalTrials.gov Identifier: NCT04293458).

## Liquid biopsy

Alternative non-invasive approaches for BO detection include the use of liquid biopsy whereby BO or early cancer-related biomarkers are assessed via an individual’s peripheral blood or breath samples. Blood or breath sampling is highly feasible and minimally invasive and is therefore more likely to improve acceptability and tolerability of BO screening. However, the most pressing challenge with these approaches is the ability to achieve accurate sensitivity and specificity whilst minimising false positives as well as the need to depend on further tests to confirm BO/early cancer diagnosis.

### Circulatory miRNAs and DNA analysis

MicroRNAs (miRNAs), small single-chain non-coding ribonucleic acids (RNAs) (18–25 nucleotides long) responsible for the regulation of physiological processes, have been investigated as biomarkers for BO/early OAC detection as both tissue-based and blood-based biomarkers [64]. The first circulatory miRNA expression profiling study in BO and OAC patients identified a panel 4 miRNA targets (miRNA-95-3p, miRNA-136-5p, miRNA-194-5p and miRNA-451a). Further validation in the same study yielded AUC of 0.832 (95% CI 0.698 – 0.967) with 78.4% (95% CI 61.8–90.2) sensitivity and 85.7 % (95% CI 57.2–98.2) specificity reported [65]. miR-143, miR-194 and miR-215 are also upregulated in the serum of BO patients in comparison to patients with non-metaplastic esophagitis [66]. Recently, miR-130a [67] as well as miR-320e and miR-199a-3p [68] have also been identified in BO. Despite the increasing number of BO-related circulatory miRNAs discovered in current literature, the lack of validatory studies on these targets have limited their clinical use for BO screening. Moreover, a recent study investigating differential miRNA signatures for BO and OAC reported a discordance between serum and tissue miRNA profiles [69]. In contrast to tissue-borne miRNAs which demonstrated an accuracy of 80% in classifying samples into normal, BO/GERD or LGD/OAC, the study failed to identify serum miRNA signature of disease state. This may limit the use of circulating miRNAs for early disease screening.

Circulating tumour DNA (CtDNA), often coupled with methylation and proteins from the plasma, is another minimally invasive method for detecting, studying and monitoring cancer. Although great progress is being made using the approach for a pan-cancer early detection test [70], it is unclear if sufficient sensitivity can be reached for early OAC screening considering that premalignant BO and dysplastic lesions are usually encapsulated within an intact basement membrane. Hence, it may not be biologically feasible to detect pre-invasive lesions using these technologies but it is too soon to draw conclusions.

### Volatile organic compounds in exhaled breath samples

Evaluation of volatile organic compounds (VOCs) in exhaled breath samples is another non-invasive approach shown to hold promise for diagnosis of various cancer types [71]. A systematic review and pooled analysis of 63 published VOC breath tests for cancer reported an AUC of 0.94, sensitivity of 79% (95% CI 77–81%) and specificity of 89% (95% CI 88–90 %) [72]. However, 41 of the studies analysed in the aforementioned review did not explore the role of cancer stage on the performance of the breath test. The other 22 studies on the other hand reported inconsistent findings.

The two main techniques currently adopted for breath sample analysis in BO/OAC patients include chemical analytical techniques, like gas chromatography-mass spectrometry [73, 74] and the electronic nose device (e-nose) (Aeonose, The eNose Company, Zutphen, Netherlands), which uses a 3 metal-oxide sensor array [75, 76]. Chemical analytical techniques are however generally more expensive and labour intensive whereas the e-nose is a more portable device with the potential for obtaining real-time breath analysis. A recent proof-of-concept study involving 402 individuals (BO patients = 129, GORD patients = 242 and control group = 132) reported the patient acceptability rate of the e-nose device to be 91.4% and demonstrated the possibility of accurately detecting BO in patients with and without GORD using VOC breath analysis with the e-nose device [76]. The VOC-based model developed and cross-validated in this study generated an AUC of 0.91 (95% CI 0.87–0.94) with 91% (95% CI 84–95%) sensitivity and 74% (95% CI 69–79%) specificity reported for BO prediction. More validation studies in larger independent cohort are warranted to confirm these promising results.

## Conclusion

Current screening guidelines for BO recommend against mass screening since the standard method for BO diagnosis and monitoring, OGD is invasive and expensive thus making it an inappropriate screening tool for a relatively uncommon cancer like OAC. This is even a more pressing issue considering that the ongoing pandemic has resulted in limited access to diagnostic services.

Most guidelines advocate for targeted screening approach whereby individuals who are most at risk of developing BO are subjected to endoscopic screening and then surveillance if warranted. However, this approach is not impacting on the high mortality from OAC. In order to improve this situation, a new clinical pathway designed to systematically identify the at-risk population for BO has to be set in place. Furthermore, cost-effective, less invasive screening tools are needed to improve accessibility to screening opportunities. Indeed, great strides are being made in these two areas. Cost-effective, easily accessible risk prediction algorithms have for instance been tested as a modality for identifying and enriching the population for BO screening. Although the majority of algorithms developed thus far have reported fair AUC’s, they may still be capable of serving enrichment purposes when used in adjunct with other more accurate screening tools. More prospective evaluation of such algorithms is required.

As regards endoscopy alternatives, newer generation of imaging techniques like TCE and OCE have undergone a series of modifications to improve portability and reduce operator dependence increasing their practicality for mass screening. Considering that biopsy sampling is limited with these technologies, in-depth assessment of the diagnostic accuracy of these tests in primary care setting is warranted. Non-endoscopic cell collection device coupled with biomarker analysis is another promising area with randomised controlled trial level evidence to support the utility of Cytoposnge-TFF3 to identify BO.

Combining all the different areas of screening discussed thus far, a three-tier precision cancer prevention-based screening programme whereby (i) easily accessible BO risk assessment algorithms are used for population-wide first-stage screening, (ii) more accurate, cost-effective and minimally invasive tools like TNE/OCE, non-endoscopic cell collection devices or liquid biopsies are used to further triage test the at-risk population in primary care setting, and (iii) suspected cases of BO or early cancer are subsequently followed up using standard endoscopy, could improve the effectiveness of the screening process. By following such a regimen, screening would be made accessible to a larger pool of individuals in an affordable manner thus reducing the number of BO cases that are missed by current practises.

With the ongoing debate on the benefit of BO surveillance, there needs to be studies evaluating the impact of BO screening using these new technologies on overall disease mortality. Furthermore, the low progression rate of BO as well as associated psychological stress amongst many other negative consequences of BO diagnosis reinforce the need for efforts to continuously be made on developing robust biomarker panel that can risk stratify BO patients. Only when this is reached would we be able to unlock the full potential of BO screening for improved clinical outcomes of OAC.
